# A randomised on-line survey exploring how health condition labels affect behavioural intentions

**DOI:** 10.1371/journal.pone.0240985

**Published:** 2020-10-26

**Authors:** Rae Thomas, Mark T. Spence, Rajat Roy, Elaine Beller

**Affiliations:** 1 Institute for Evidence-Based Healthcare, Bond University, Gold Coast, Australia; 2 Bond Business School, Bond University, Gold Coast, Australia; University of Nottingham School of Medicine, UNITED KINGDOM

## Abstract

**Objectives:**

We examined the effect of ‘labels’ versus ‘descriptions’ across four asymptomatic health conditions: pre-diabetes, pre-hypertension, mild hyperlipidaemia, and chronic kidney disease stage 3A, on participants’ intentions to pursue further tests. There were four secondary objectives: 1) assessing confidence and satisfaction in their intention to test further; 2) revealing psychological drivers affecting intentions; 3) exploring whether intentions, confidence and satisfaction differ by label vs. description and health condition; and 4) producing a perceptual map of illnesses by label condition.

**Methods:**

Practitioner validated health-related scenarios were used. Two variants of each condition were developed. Participants were recruited through Qualtrics from Australia, Ireland and Canada and randomly assigned two ‘labelled’ or two ‘descriptive’ scenarios.

**Results:**

There was no significant difference in intentions to test between label and description conditions (95% CI -0.76 to 0.33 points, *p* = 0.4). Confidence and satisfaction were both positively associated with intentions: regression coefficient (β) for confidence β = 0.58 points (95% CI 0.49 to 0.68, *p* < .001) and for satisfaction 0.67 points (95% CI 0.57 to 0.77, *p* < .001). Predisposition to seek healthcare (β = 0.72; 95% CI 0.47 to 0.98), attributing illness to bad luck (β = -0.16 points; 95% CI -0.3 to -0.02), and concern about the health condition (β = 0.51; 95% CI 0.38 to 0.65) also significantly predicted intentions.

**Conclusions:**

Unlike studies investigating symptomatic illnesses, the disease label effect on behavioural intentions was not supported suggesting that reducing demand for medical services for borderline cases cannot be achieved by labelling. The average intention to test score was higher in this sample than previous symptomatic health-related studies and there was a positive relationship between increased intentions and confidence/satisfaction in one’s decision. Exploratory insights suggested perceptions of the four labelled asymptomatic illnesses all shifted toward greater levels of dread and concern compared to their respective description condition.

**Trial registration:**

ACTRN12618000392268.

## Introduction

How and what are communicated between health care providers and consumers has ramifications. Much research has shown that how information–medical or otherwise–is presented to a decision maker profoundly affects their decision making [1–4]. Continuing the line of research by scholars such as Scherer et al. [5], Copp et al. [6] and Nickel et al. [7], we examined the effects of including versus excluding the health condition name (e.g., informing the patient that they might have ‘pre-diabetes’ versus ‘a slight elevation in your blood sugar level’) on decision making and explored how individual characteristics may impact these decisions.

Unlike prior research that studied symptomatic illnesses [5–7], we focused on four asymptomatic health conditions: pre-diabetes, pre-hypertension, mild hyperlipidaemia, and chronic kidney disease stage 3A (CKD 3A). Each has been the focus of controversy due to diagnostic threshold changes and the potential of overdiagnosis [8–11]. Widening disease definitions and reducing thresholds narrows the divide between being considered healthy or unwell, and ultimately increases the number of individuals classified as abnormal [12]. Therefore, how we communicate test results close to these thresholds may be important as it could affect patient’s psychological states (such as increasing or decreasing anxiety) as well as impose increased demands on the healthcare system (pursuing questionable further tests/interventions).

Previous research suggests using medical labels to describe health conditions increases individuals’ intentions to pursue further treatment [7]. For example, Scherer et al. [5] showed that providing the name of a commonly occurring child health condition (gastroesophageal reflux disease [GERD]) to mothers rather than simply describing the symptoms interacted with the reported efficacy of medication. Disconcertingly, compared with the symptom description group, mothers who were given the scenario informing them of the name of the condition (GERD) indicated that they would be more likely to seek medicative treatment even when informed of the medication’s ineffectiveness. Similarly, Copp et al. [6] found that the presence versus absence of a health condition label (polycystic ovary syndrome [PCOS] versus hormonal imbalance) in their hypothetical scenario study affected the likelihood of an individual wanting an ultrasound. In the presence of the PCOS label, participants reported that they would be more likely to have an ultrasound, thought the condition was more severe, and reported lower self-esteem compared with the symptom description group. However, providing information on the potential for overdiagnosis, due to increased sensitivity of ultrasounds, decreased participants’ intentions and perceived severity in both groups. Recently, a systematic review [7] that included these two studies and five others concluded that the presence of a health label increased the likelihood of invasive treatment, heightened anxiety and perceptions of illness severity compared with no label.

This study investigates whether the label effect applies to asymptomatic health conditions where individuals are on the borderline of being classified as having these conditions. The current study design mimics Scherer et al. [5] and Copp et al. [6] in that we too used hypothetical scenarios. However, four issues differentiate this research effort from preceding studies. First, prior studies [7] examined situations where participants were provided scenarios of health conditions with a description of symptoms the participant might experience or used scenarios that did or did not include the word ‘cancer’. Scholars have suggested that the use of terms like ‘cancer’ should be redefined and its use circumscribed because such terms “corrupt thought” [13]. Here, we study whether labelling asymptomatic illnesses affects intentions to pursue further tests. This is the primary research outcome. Second, external validity was enhanced two ways: we studied four common health conditions using samples from three countries considered broadly similar. In the absence of experiencing any symptoms, does the “labelling effect” still hold? Third, we examined potential psychological drivers that could be affecting one’s decision to (or not to) pursue further medical treatment. Fourth, there is some evidence that disease labels (health condition name included) and description only scenarios affect perceptions of severity [6], but findings are equivocal [5]. Therefore, in addition to examining the differences in intention to undertake further tests between labelled and description only scenarios (primary research objective), exploratory analysis was undertaken to see if labelling affected perceptions of risk for each health condition.

## Methods

### Participants

Participants were recruited in March 2018 through Qualtrics (www.qualtrics.com). Qualtrics uses existing, nationally representative panels of individuals who have previously agreed to complete surveys. Participants were required to read an Explanatory Statement that included describing the purpose of the study and that participation was voluntary, that they could discontinue at any time, and that names would not be collected. The survey would not advance without all questions on each page answered, including attention check questions, hence all returned surveys were complete. Participating in the on-line survey was accepted as informed consent. Participants were eligible if they were 45 years or older because the health conditions examined are more prone to afflict older adults. All participants resided in either Australia, Canada or Ireland, countries with similar healthcare models. There were no other restrictions to participation.

### Procedure

**Fig 1:**
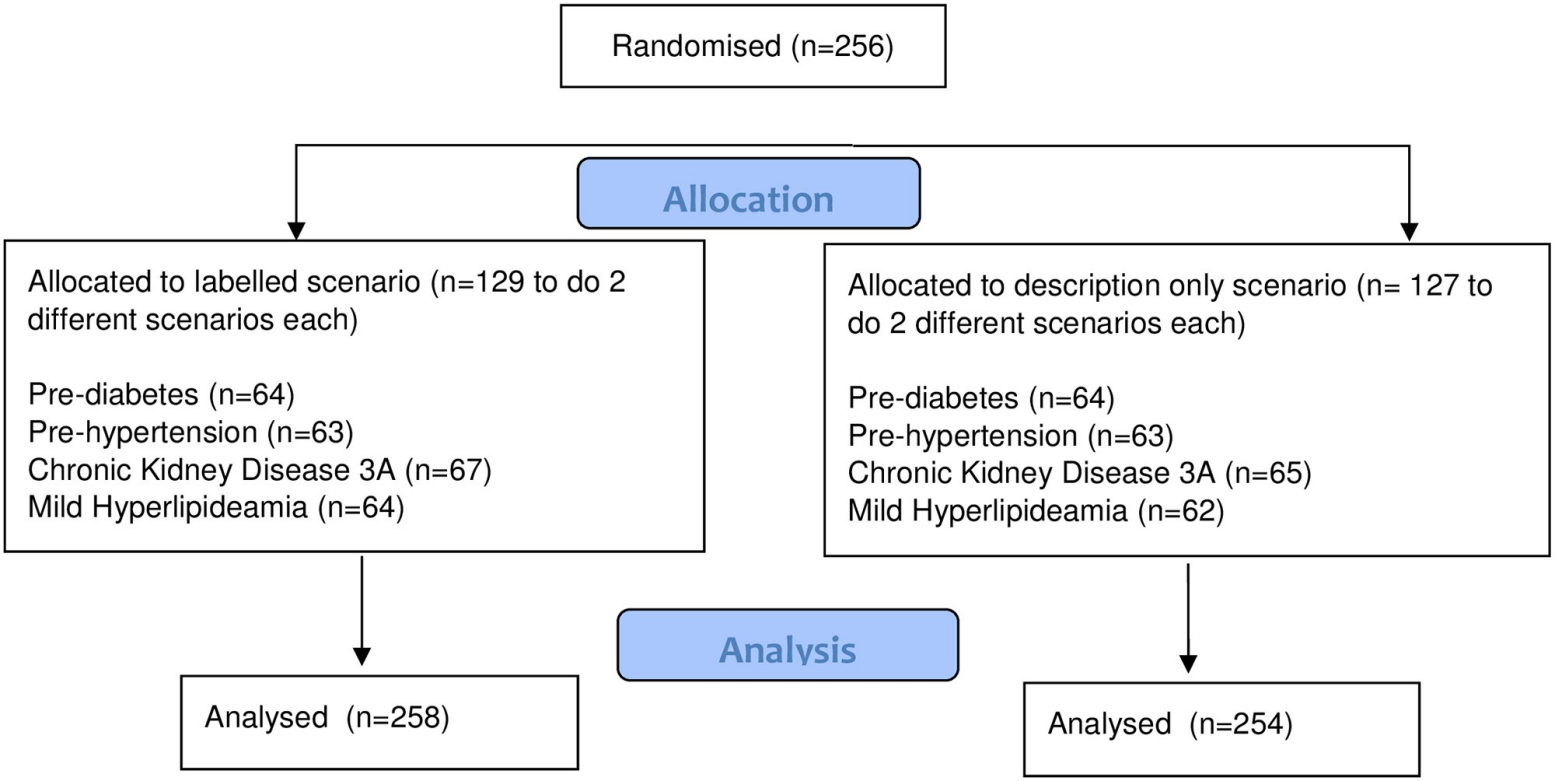
Consort flow diagram.

All participants completed demographic questions and a series of psychological measures prior to reading two health-related scenarios. Participants were then randomly assigned by Qualtrics to read either two “labelled” or two “descriptive” hypothetical scenarios (Fig 1). Scenarios described the outcome of a recent health test using either the medical terms (labelled scenario) or descriptive terms (descriptive scenario). There were “labelled” and “descriptive” scenarios for four health conditions with controversies surrounding their threshold cut-offs (pre-diabetes [8], pre-hypertension [9], mild hyperlipidaemia [10], and CKD 3A [11]). All scenarios were developed in consultation with primary health practitioners unrelated to this project. See Appendix for scenarios and references for threshold criteria. Within each scenario, information about the potential for overdiagnosis for the specific health condition was provided as were lifestyle behaviours known to reduce the likelihood of developing each condition. Following the scenarios, participants were asked questions on risk perception, stigma, the likelihood of attending follow-up tests for the specific condition, and the level of their confidence and satisfaction with their decision. Bond University Human Research Ethics Committee (#16123) provided ethics approval.

### Measures

The primary outcome was assessed using a single item similar to Copp et al.: “Which best describes your intention to have the follow-up tests described in the scenario next week?” [6]. Responses ranged from 1 (definitely will not) to 10 (definitely will). With reference to their intentions, we also asked, “How confident are you with your decision in the above scenario?” and “How satisfied are you with your decision in the above scenario?” (1 = not at all confident [satisfied] and 7 = very confident [satisfied]). These latter two measures allowed testing of the first of the four secondary objectives.

Additional secondary analyses included measuring a series of potential psychological drivers that could affect intentions, confidence, and satisfaction. To achieve this, perceptions of health control were assessed using the 18-item multidimensional health locus of control, [14, 15] (MHLOC). Subscales of internal, external, and chance were created by summing related items. Regulatory focus was assessed using the 10-item Composite Regulatory Focus Scale [16] scored as two subscales, Promotion Focus and Prevention Focus. Responses for both scales ranged from 1 (strongly disagree) to 6 (strongly agree) with higher scores favouring stronger subscale influence. Individuals are predisposed to a promotion versus prevention self-regulatory orientation, with the latter placing relatively more emphasis on responsibilities and security needs [17].

Participant’s self-perceptions toward their medical use was measured using the Medical Maximizer-Minimizer Scale [18] (MMMS). Responses were averaged to form a total score. Higher scores suggest a tendency toward more frequent health service use. The Ten Item Personality Inventory [19] (TIPI) was used to assess individual extraversion, agreeableness, conscientiousness, emotional stability, and openness to experience (2 items each). Items on both MMMS and TIPI were scored on a 7-point scale ranging from 1 (strongly disagree) to 7 (strongly agree).

To assess risk orientation in health behaviours, we used five health/safety items described by Weber and colleagues [20]. Response options ranged from 1 (extremely unlikely) to 5 (extremely likely).

After reading the scenario, subjects reported their perceptions of the risks associated with the specific health condition depicted in the scenario. Risk perceptions were measured with six items adapted from Fox-Glassman and Weber [21]. Items focused on perceptions of individual responsibility versus bad luck, known versus unknown risks, common versus dreaded condition, immediate versus delayed risk, uncontrollable versus controllable condition, and unconcerned versus concerned. Responses were rated on 7-point scales. Participants also reported their perceptions regarding stigma associated with the conditions (comfort, embarrassment, social isolation, and concealment) on four 7-point scales adapted from the literature [22].

### Statistical analyses

Differences in intention to undertake further tests between labelled and description only scenarios is the primary research outcome, hence power analysis relates to this variable when data are collapsed across the four health conditions. Copp et al. [6] reported a statistically significant between group difference of 0.86 points in intention score, SD 2.7. For sample size calculations, we considered a more conservative 1-point difference in the 10-point future intentions to test scale to be the smallest clinically meaningful difference. With type II error at 0.2 and statistical significance set to *p =* 0.05, a power calculation indicated 115 participants were needed per group (230 total). The Power and Sample Size software (PS) was used for the calculation, based on an independent t-test and continuous response measure [23]. To ensure equal numbers from each country, we aimed to recruit 240 participants (80 per country).

The primary outcome of the difference in intention scores for labelled and description-only scenarios was analysed using mixed models linear regression rather than a simple t-test since we had two scenario responses per person, nested within participant. Participants were fitted as a random effect.

For secondary outcomes, we used the same regression method as above. P-values were not adjusted for multiple comparisons. First, we report differences in satisfaction and confidence in participant’s decision to undertake further tests between labelled and description only scenarios. Second, we conducted mixed methods regression for each of the proposed psychological measures. Time to read and respond to the medical scenario was also considered because time is a proxy that captures depth of information processing [24]. Significant variables from univariate regression models (levels of significance set at *p =* 0.1) were then included in multivariable regression analyses using backwards stepwise elimination to examine the effect of participant characteristics and specific perceptions of each health condition on an individual’s intention to pursue follow-on tests. In an additional step, we considered the interaction of each of these variables and label versus descriptive scenarios to assess if any of the interactions modify the effect of labelling on intention to test. Third, we conducted an exploratory analysis of whether intentions to pursue follow on tests as well as confidence and satisfaction differ by health condition. These were exploratory analyses given the smaller sample sizes at the health condition level. For all regression models, assumptions concerning linearity, homoscedasticity, and independence and normality of residuals were checked.

Finally, also as an exploratory analysis, we produced perceptual maps of the four health conditions comparing labelled and description only conditions based on participants’ responses to six risk perception measures. Principle component analysis with varimax rotation and Kaiser normalization was used to reduce dimensions. The resultant factor scores were used to generate Y and X coordinates on a perceptual map. Respondent factor scores were created using a regression approach. This required two steps: 1) standardized values for the respondent’s answers to the six risk perception measures were computed; and 2) these standardized values were then multiplied by the values in the Component Score Coefficient Matrix. The resultant factor scores were standardized (M = 0, SD = 1). The average of the factor scores was then computed for each of the eight conditions (label/description by four illnesses) and for each of three factors. See S2 and S3 Tables in S1 File. All data were analysed in STATA 14 and SPSS Statistics 23.

## Results

Participants (N = 256) were recruited from Australia (n = 87), Ireland (n = 82), and Canada (n = 87). Participants were aged between 45 and 82 years (Mean = 56.5 years, SD = 8.2; Table 1) with an even gender distribution (males, 51%; females 49%). Two labelled scenarios were randomly allocated to 129 individuals and two description only scenarios randomly allocated to 127 participants (Fig 1). On average, participants scored themselves 6.6/10 for overall health and relatively high on predisposition to use healthcare (MMMS, mean = 45.8/70) agreeableness, and conscientiousness (both mean = 10.7/14). There was no statistically significant country of origin or gender effects on intentions to pursue further tests, nor did they improve precision, hence these potential covariates were excluded from further analyses. Overall, participants scored similarly between groups (Table 2 and S1 Table in S1 File).

**Table 1:** Participant demographics and characteristics (N = 256).

	Mean / Freq	SD / (%)	Min	Max
Age	56.5	8.2	45	82
Gender				
Male	130	(50.78)		
Female	126	(49.22)		
Country				
Australia	87	(33.98)		
Canada	87	(33.98)		
Ireland	82	(32.03)		
Overall Health (scored /10)	6.6	1.9	1	10
MHOL (scored /36)				
External	23.2	4.1	11	35
Internal	23.6	4.8	6	36
Chance	17.4	5.1	6	35
Regulatory Focus (scored /30)				
Promotion	19.8	3.4	7	29
Prevention	19.1	3.3	9	29
Medical Minimizer/Maximizer Total (MMMS scored /70)	45.8	9.2	17	67
Personality (scored /14)				
Extraversion	7.4	2.5	2	14
Agreeableness	10.7	2.1	3	14
Conscientiousness	10.7	2.1	4	14
Emotional Stability	9.2	2.5	2	14
Openness	9.8	2.2	2	14
Risk Orientation (scored /35)	13.7	3.8	8	24

**Table 2:** Unadjusted means and standard deviations for risk perception, stigma, intention to test, confidence and satisfaction across grouped description only and labelled scenarios.

	Description only Scenarios (n = 254)		Labelled Scenarios (n = 258)	
	Mean	SD	Mean	SD
Risk Perception (score 1–7)				
Responsibility vs Luck	3.2	1.3	3.2	1.3
Known vs Unknown risks	3.8	1.6	3.6	1.6
Common vs Dreaded Condition	3.1	1.3	3.4	1.5
Immediate vs Delayed risk	4.7	1.5	4.6	1.5
Uncontrollable vs Controllable Condition	5.4	1.3	5.6	1.3
Unconcerned vs Concerned	4.3	1.4	4.4	1.5
Stigma (score 1–7)				
Comfort	5.3	1.5	5.5	1.4
Embarrassment	2.3	1.5	2.1	1.4
Social Isolation	2.4	1.5	2.4	1.6
Concealment	5.2	1.6	5.5	1.5
Intention to conduct future test (score 1–10)	7.3	2.3	7.1	2.7
Confidence in decision (score 1–7)	7.7	2.0	8.0	1.9
Satisfaction in decision (score 1–7)	7.9	2.0	8.1	1.9

### Primary outcome—Differences between ‘label’ compared with ‘descriptive only’ scenarios and intentions to test further

Individuals who read labelled scenarios scored an average of 0.22 points lower on the intentions to undertake further tests measure (scale 1 to 10) than participants who read description only scenarios, but this was not statistically significant (95% CI -0.76 to 0.33 points, *p* = 0.4). This contradicts the prevailing view that there is a labelling effect [5–7]; but unlike prior studies, the focus here is on symptomatic illnesses.

### Secondary outcomes

Caution should be made when interpreting our secondary outcomes as the study was powered for the primary outcome and consequently all these outcomes are underpowered.

### Differences in intention scores by health condition

After adjusting for labelled and description only and two scenarios per person, there was no significant difference in intention scores between the four health conditions. Participants who read scenarios suggesting pre-hyperlipidaemia (regardless of whether it was labelled or description only), scored on average 0.37 points lower on intentions to test than individuals who read pre-diabetes scenarios, but this difference was not statistically significant or clinically important (Table 3). Analyses at the individual illness level are secondary outcomes and consequently underpowered, and thus must be interpreted with caution.

**Table 3:** The effect of labelling health conditions on an individual’s future intention to test.

	Mixed models linear regression			
	Coeff.	95% CI		*p*
Labelled scenario	-0.22	-0.76	0.33	0.43
Condition				
Pre-diabetes (reference)				
Pre-hypertension	0.38	-0.41	0.48	0.87
Chronic kidney disease stage 3A	0.09	-0.37	0.54	0.71
Mild hyperlipidaemia	-0.37	-0.80	0.06	0.09

#### The relationship between an individual’s intention to test and their confidence and satisfaction with their decision

Confidence and satisfaction in decisions were both associated with a higher intention to conduct further tests both overall and when adjusted for labelling. Overall, for each point higher in confidence, participants scored 0.58 points higher in intention to test (95% CI 0.49 to 0.68, *p* < .001) and an increase in satisfaction was also associated with an increase in intention scores by 0.67 points (95% CI 0.57 to 0.77, *p* < .001). Confidence and satisfaction remained significant after adjusting for labelling (0.59 points higher in intention to test for confidence, 0.67 points for satisfaction). Although statistically significant, these differences were not more than 1 point in the intention to test scale which we deemed clinically meaningful. There was no difference in confidence or satisfaction scores when comparing the same health condition (e.g., pre-hypertension either labelled or pre-hypertension descriptive only scenario) or when the specific health condition was adjusted for labelling/description only (e.g., pre-hypertension labelled scenario compared with pre-hypertension descriptive only scenario).

#### Effects of psychological characteristics, risk perceptions and stigma on intentions to conduct further tests

To explore the effects of psychological characteristics, perceptions of risk, stigma related to the health condition and response time, we conducted a mixed model regression, adjusting for labelling. We also included all interactions between these variables and label versus description only to test if labelling was an effect modifier. All main effects are reported in Table 4. In the multivariable model, three variables contributed to predicting an individual’s intention to conduct further tests; no interactions were significant. For every one-point higher in an individual’s predisposition to seek health care, their intention score increased 0.72 points (95% CI 0.47 to 0.98). The more a person considered the health condition was due to bad luck (compared with personal responsibility) their intention score decreased by 0.16 points (95% CI -0.3 to -0.02). Finally, the more concerned (compared with unconcerned) the individual was about the health condition, their intention to conduct further test score increased by 0.51 points (95% CI 0.38 to 0.65).

**Table 4:** The effects of participant characteristics, risk perception and stigma to intention to test scores after adjusting for labelling.

	Univariable Analyses				Final Multivariable Analyses			
	Coeff.	95% CI		*p*	Coeff.	95% CI		*p*
Overall Health	0.14	-0.01	0.28	0.06				
MHOL								
External	0.67	0.28	1.06	0.001				
Internal	0.13	-0.21	0.47	0.462	-	-	-	-
Chance	-0.16	-0.48	0.16	0.332	-	-	-	-
Regulatory Focus								
Promotion	0.47	0.08	0.87	0.02				
Prevention	-0.06	-0.48	0.36	0.78	-	-	-	-
Predisposition to seek health care (MMMS)	1.03	0.76	1.30	< .001	0.72	0.47	0.98	< .001
Personality								
Extraversion	0.20	-0.01	0.41	0.07	-	-	-	-
Agreeableness	-0.03	-0.29	0.23	0.82	-	-	-	-
Conscientiousness	0.19	-0.07	0.45	0.16	-	-	-	-
Emotional Stability	-0.08	-0.30	0.14	0.48	-	-	-	-
Openness	0.14	-0.10	0.38	0.27	-	-	-	-
Risk Orientation	0.07	-0.29	0.43	0.69	-	-	-	-
Risk Perception								
Individual responsibility vs Bad luck	-0.27	-0.42	-0.11	0.001	-0.16	-0.30	-0.02	0.03
Known vs Unknown risks	0.01	-0.11	0.13	0.85	-	-	-	-
Common vs Dreaded Condition	0.11	-0.03	0.24	0.12	-	-	-	-
Immediate vs Delayed risk	-0.36	-0.49	-0.23	< .001				
Uncontrollable vs Controllable Condition	0.04	-0.13	0.21	0.63	-	-	-	-
Unconcerned vs Concerned	0.62	0.49	0.76	< .001	0.51	0.38	0.65	< .001
Stigma								
Comfort	-0.02	-0.17	0.13	0.83	-	-	-	-
Embarrassment	0.00	-0.16	0.15	0.96	-	-	-	-
Social Isolation	0.04	-0.10	0.18	0.61	-	-	-	-
Concealment	0.05	-0.09	0.19	0.49	-	-	-	-

### A perceptual map of health conditions by label condition

We conducted principle component factor analyses on particpant’s responses to six risk perception measures. Three dimensions emerged that were used to produce perceptual maps that illustrate the health conditions when scenarios were labelled versus described. Three measures loaded onto Factor 1, capturing perceptions of the condition’s dreadfulness (compared with common), its immediacy (compared with delayed) risk and concern (compared with unconcerned) about the health condition. Higher numbers indicate higher levels of these characteristics. Two measures loaded onto Factor 2: whether the health condition was due to bad luck (versus individual responsibility) and whether the risks were known or unknown. Factor 3 reflects one measure, whether the condition is controllable: higher numbers indicate that through diligence the individual can avoid the health condition. Scores for the rotated component matrix are reported in S2 Table in S1 File.

**Fig 2:**
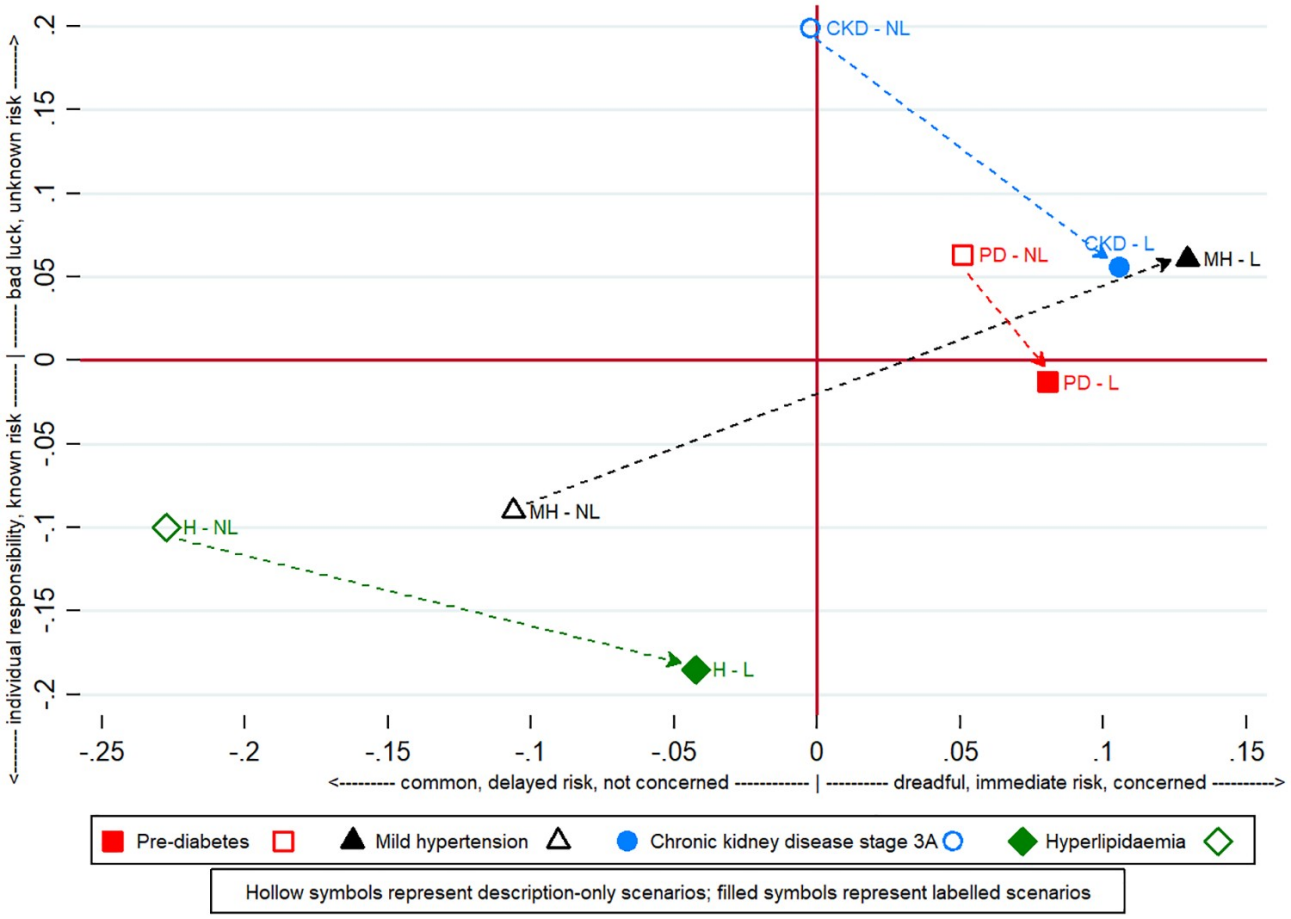
Perceptual map for risks associated with label versus descriptive diseases.

Fig 2 illustrates one of the three two-dimensional plots that could be created from the factor scores. The figure illustrates the intention to test means for each of the four health conditions (two for each condition) based on respondents’ factor scores for common condition, delayed risk, and not concerned versus dreadful condition, immediate risk, and concerned (Factor 1, horizontal axis) and individual responsibility and known risk versus bad luck and unknown risk (Factor 2, vertical axis). This analysis, being at the individual health condition level rather than the group level, is underpowered hence exploratory; nevertheless, it is interesting that across all four health conditions the presence of the label (filled symbols) appears to induce higher perceptions of dread than symptom only descriptions (relative to their corresponding description condition, all had higher values on Factor 1). Also, for mild hyperlipidaemia, CKD 3A and pre-diabetes, but not pre-hypertension, there appears a move toward more responsibility and known risk when labels for the health condition are used compared with description only scenarios (i.e., lower values on Factor 2). The factor scores by health conditions are reported in S3 Table in S1 File.

#### Post hoc analyses comparing individual health conditions on intention to undertake future tests

Additional exploratory analyses were conducted at the individual illness level and are subsequently underpowered. Perceptions of risk differed between health conditions and were associated with intentions to pursue further tests (Table 5). Using risk perceptions associated with pre-diabetes as the baseline, participants who read CKD 3A scenarios had intention to test scores 0.50 points higher (95% CI 0.17 to 0.82, p = 0.003) for each point they perceived the condition was contracted more through bad luck than personal responsibility. However, for CKD 3A, participants had lower intention to test scores for each point they thought the condition was controllable (compared with uncontrollable, -0.46 points 95% CI -0.77to -0.15, p = .004). Taken together, these suggest when participants read scenarios about CKD 3A, they score higher on intentions to test (compared with pre-diabetes) when they perceived CKD 3A was contracted through bad luck, but when the CKD 3A was considered controllable participants were less likely to have future tests compared with pre-diabetes.

**Table 5:** The effects of health condition and risk perception on intention to undertake future tests.

	Pre-Hypertension			Chronic Kidney Disease 3A			Mild Hyperlipideamia		
	Coeff.	95% CI		Coeff.	95% CI		Coeff.	95% CI	
Pre Diabetes (reference)									
Risk Perception									
Individual responsibility vs Bad luck	0.28	-0.04	0.60	0.50*	0.17	0.82	-0.06	-0.39	0.26
Known vs Unknown risks	0.23	-0.16	0.62	0.33	-0.06	0.73	0.09	-0.31	0.48
Common vs Dreaded Condition	0.22	-0.12	0.55	0.04	-0.30	0.38	-0.30	-0.64	0.04
Immediate vs Delayed risk	-0.04	-0.41	0.34	0.16	-0.22	0.53	0.32	-0.06	0.69
Uncontrollable vs Controllable Condition	-0.56*	-0.87	-0.26	-0.46*	-0.77	-0.15	-0.14	-0.45	0.17
Unconcerned vs Concerned	-0.23	-0.58	0.12	-0.84	-0.44	0.27	-0.32	-0.67	0.04

* *p* < 0.05

Similarly, compared with pre-diabetes scenarios, for participants reading the pre-hypertension scenarios intention to test scores were 0.56 points lower (95% CI -0.87 to -0.26, p < .001) for each point they perceived the condition was controllable (compared with uncontrollable). No other risk perception measures differed between health conditions. After adjusting for labelling, the effect on intention to test for both perceptions of individual responsibility vs bad luck and controllable vs uncontrollable were still significantly different for pre-hypertension and CKD3A.

## Discussion

By focussing on asymptomatic conditions and using an international sample we have expanded the research on medical labelling. Previous “labelling” studies have focussed on symptomatic conditions [5, 6] or on language such as various terminologies for papillary thyroid cancer [25], ductal carcinoma in situ [26] or bone fractures [27]. Overall, these studies found an increased tendency to pursue further or more invasive testing when medical labels or the term cancer were provided compared with a description of the symptoms only or lay terminology. Contrary to these studies, we did not find a statistically significant difference between participant’s intention to test scores when presented with either labelled or descriptive only scenarios. The illnesses studied were specifically chosen because of recent diagnostic threshold changes and the potential of overdiagnosis [8–11]. A greater number of individuals are therefore likely to be classified as abnormal [12], and their potential inclination to pursue further testing could strain health care systems. We advance several possible explanations why our findings regarding intentions to pursue further testing differ.

In contrast to prior studies that explored symptomatic scenarios or the use of cancer terminology, we compared label with description only scenarios for four different *asymptomatic* health conditions (pre-hypertension, mild hyperlipidemia, pre-diabetes, and CKD 3A) and participants were told their test results were “close to the threshold”. These conditions are common and thus potentially familiar to participants (aged 45+) who may have habituated to routine health checks and taking follow-up tests. Although individuals were randomly selected in the age range most affected by the health conditions in the scenarios, our participants self-reported as high users of health services and rated themselves highly on agreeableness and conscientiousness, which may increase their testing practices. Their generally high scores on the medical minimizer maximiser scale (mean = 4.6/7) suggests that participants preferred to undertake interventions or medications they perceived positively and are biased toward action rather than inaction or “watchful waiting” [18]. Interestingly, the average intention to test score in the current study was 7.3 for description only compared with 7.1/10 for labelled scenarios. This is higher than the intentions scores in the polycystic ovary syndrome study (6.6 and 5.8/10 in the label versus description condition) [6]. It may also be the case that given the age of participants, some are familiar with and possibly taking medication for the conditions studied. We did not ask participants about pre-existing health conditions or about familiarity of the health condition assigned. We also did not control for socio-economic status and health literacy. However, participants were randomly assigned to conditions, so it would be unlikely that levels of familiarity, socio-economic status, and health literacy would systematically vary across conditions. Participants were asked to imagine themselves as the recipient of the healthcare information, however this is standard practice in these experimental designs. Individuals on Qualtrics panels who are routinely exposed to surveys may also be more conscientious than individuals randomly selected by other means.

Using participants from survey panels may introduce selection bias as participants are reimbursed in some way for their time. However, reimbursing participants is common and many studies have relied on recruitment agencies [e.g., 26, 28]. Recruiting participants in this manner affords a broad geographical reach increasing the study’s generalisability and tests the “labelling effect” with a large cross-cultural sample.

We used practitioner validated scenarios that also provided information on alternatives to testing and information on overdiagnosis. However, these scenarios were not tested for literacy levels. For clarity and consistency, the scenarios were lengthy and worded similarly; however, by doing this we may have provided too much information. Outside the medical domain, “information overload” has been shown to lead to a reduction in decision making effort and quality [29].

In mediation analysis it is customary to have the manipulation and measure the effects of interests, then measure potential effect modifiers. We reversed that order. We purposely positioned the psychological measures such as MMMS at the start of the survey because we wanted participants to answer these without seeing the scenarios. However, because the characteristics were health-related we may have inadvertently primed our participants to think about their health which in turn may have impacted their responses.

We found that confidence and satisfaction in a person’s decision to undertake further tests were both positively associated with increased intentions to test which held when adjusted for labelling. These findings suggest that irrespective of whether the health condition was given a medical label or not, those that were more prone to pursue further tests also felt more confident and satisfied with their decision. The lack of independence between intentions and confidence/satisfaction is disconcerting and merits further research. It is reasonable to assume clinicians would like their patients to be confident and satisfied with their decisions without having to resort to increasingly active treatment.

Another unique element of this study included our exploration of risk perceptions and effect modifiers. Although not statistically significant and we acknowledge that analyses at the individual illness level are underpowered, the illustrative risk perception figure shows that medical labels appear to evoke greater dread and concern compared with a description of symptoms. This is consistent with a recent narrative synthesis of medical terminology [7] where medicalised terms evoked greater perceived severity of the health condition. The emerging pattern in our risk perception figure generates new hypotheses about why this might be. Prior labelling studies have not systematically investigated this issue using existing risk perception measures [22, 23] and what insights do exist are equivocal [7]. Insights revealed from these exploratory analyses are therefore meant to stimulate follow-on research.

Finally, although other studies have found a significant difference between labelling and description only scenarios in participants’ intention to test further, prior research has not systematically endeavored to unearth why. We found participants predisposed to seek health care were more likely to test further as were those who perceived the health condition more concerning and more likely attributable to personal responsibility.

Collectively these insights lead us to conclude that how medical labels affects health decision making is more nuanced than previous studies suggest. Unlike other studies, that presented participants with scenarios that either described the health condition or ascribed a medical label [7], our results did not show a significant difference between these two scenarios. Potentially, our choices of asymptomatic health conditions compared with symptomatic conditions such as GERD [5] and PCOS [6] influenced these findings. Decisions made about whether to undertake further tests the context of asymptomatic health conditions might differ from those made for symptomatic conditions. Here, we also found the ‘default option’ to pursue further tests within this mature aged sample to be high; indeed, higher than in studies involving symptoms. Given the aging demographics in all of three countries and the prevalence of the health conditions studied, this does not bode well for already stretched medical facilities/budgets.

### Practice implications

Good communication is the cornerstone of healthcare. To address the issues of overdiagnosis and overtreatment and the burdens these create for individuals and the health care system, it is essential that health care professionals better understand how individuals respond to medical labels, the psychological mechanisms that drive their decision-making, as well as whether responses to asymptomatic health conditions differ from symptomatic ones. In this study, nudging behaviour toward watchful waiting using different labels was not effective for the asymptomatic illnesses studied.

## Supporting information

S1 File Scenarios provided to participants and the references for the described thresholds. (DOCX)

S1 Checklist CONSORT 2010 checklist of information to include when reporting a randomised trial*. (DOC)
