# Supplementary material for: A randomised on-line survey exploring how health condition labels affect behavioural intentions
Source: PLoS One. 2020 Oct 26;15(10):e0240985. doi: 10.1371/journal.pone.0240985 (PMC7588049; doi:10.1371/journal.pone.0240985)
Supplement: S1 File — (DOCX) [file pone.0240985.s001.docx]

**S1 File**

**Scenarios provided to participants and the references for the described thresholds.**

**Pre-Diabetes Labelled**

Imagine you are 47. You recently had a health check-up with your doctor. You exercise irregularly, but you think you eat well, you feel healthy and you are not overweight. Your blood test from last week said your HbA1c level (a measure of blood sugar levels) was 6.1% which your doctor said might suggest you have pre-diabetes. A current guideline suggests the test cut-off for this potential problem should be under 6%. You are close to this threshold

Your doctor said you could have another test next week to test the stability of this reading, you could have another test later in the year or you could choose not to have another test because your test level is close to the normal threshold. Alternatively, your doctor said that to help lower your blood sugar levels and improve your health overall you could increase your exercise to ½ hour walks for 5 days a week, eat more dietary fibre and eat less refined sugars and flours.

Overdiagnosis occurs in medical practice when you get a diagnosis that ends up causing you more harm than good. It happens often when a healthy person is diagnosed with a very early form of a potential health problem, but that problem would never in fact have developed to cause them any symptoms or even any health problems. Having elevated blood sugar levels can increase your chance of developing diabetes. However, evidence shows that over 60% of people with your blood sugar level do not become diabetic and so you have the potential to be overdiagnosed.

There is a risk that you may have a problem with how your body processes sugar. You could therefore go for further testing. Identifying a potential health problem early could slow progression towards a more serious problem if one was going to occur. If you choose not to take future tests, you would benefit by avoiding further costs and inconvenience associated with more testing. You might also avoid being diagnosed with a health problem that would never harm you and avoid the psychological impacts like anxiety and depression that some people experience with more testing.

**Reference for threshold cut off:** World Health Organization. Use of glycated haemoglobin (HbA1c) in the diagnosis of diabetes mellitus. abbreviated report of a WHO consultation. WHO, 2011.

**Pre-Diabetes Description Only**

Imagine you are 47. You recently had a health check-up with your doctor. You exercise irregularly, but you think you eat well, you feel healthy and you are not overweight. Your blood test from last week showed you had a slight elevation in your blood sugar level. A current guideline rates your blood sugar level as close to the threshold for a potential problem.

Your doctor said you could have another test next week to test the stability of this reading, you could have another test later in the year or you could choose not to have another test because your test level is close to the normal threshold. Alternatively, your doctor said that to help lower your blood sugar levels and improve your health overall you could increase your exercise to ½ hour walks for 5 days a week, eat more dietary fibre and eat less refined sugars and flours.

Overdiagnosis occurs in medical practice when you get a diagnosis that ends up causing you more harm than good. It happens often when a healthy person is diagnosed with a very early form of a potential health problem, but that problem would never in fact have developed to cause them any symptoms or even any health problems. Having elevated blood sugar levels can increase your chance of developing a serious health problem. However, evidence shows that over 60% of people with your blood sugar level do not develop that problem and so you have the potential to be overdiagnosed.

There is a risk that you may have a problem with how your body processes sugar. You could therefore go for further testing. Identifying a potential health problem early could slow progression towards a more serious problem if one was going to occur. If you choose not to take future tests, you would benefit by avoiding further costs and inconvenience associated with more testing. You might also avoid being diagnosed with a health problem that would never harm you and avoid the psychological impacts like anxiety and depression that some people experience with more testing.

**Reference for threshold cut off:** World Health Organization. Use of glycated haemoglobin (HbA1c) in the diagnosis of diabetes mellitus. abbreviated report of a WHO consultation. WHO, 2011.

**Pre-Hypertension Labelled**

Imagine you are 50, exercise a couple of times a week, try to eat well, and you feel healthy. You recently had a health check with your doctor. At this check up your blood tests came back with no problems, but your doctor observed that your blood pressure was 125/80 mmHg which your doctor said might suggest you have pre-hypertension. A current guideline suggests that a test cut-off for this potential problem should be less than 120-129/80 mmHg. Your blood pressure is close to the threshold.

Your doctor said you could have another test next week to test the stability of this reading, you could have another test later in the year, or you could choose not to have another test because your test level is close to the normal threshold. Alternatively, your doctor said to help lower your blood pressure and improve your health overall you could increase your exercise to ½ hour walks for 5 days a week, eat less salty foods and drink less alcohol.

Overdiagnosis occurs in medical practice when you get a diagnosis that ends up causing you more harm than good. It happens often when a healthy person is diagnosed with a very early form of a potential health problem, but that problem would never in fact have developed to cause them any symptoms or even any health problems. Having a slightly elevated blood pressure could increase your risk of having a heart attack or stroke in the next 5 years. However, 96% of people with similar blood pressure do not go on to have a heart attack or stroke within 5 years even when they don’t take medication, so you have the potential to be overdiagnosed.

There is a risk that you may have a problem with your blood pressure. You could therefore go for further testing. Identifying a potential health problem early could slow progression towards a more serious problem if one was going to occur. If you choose not to take future tests, you would benefit by avoiding further costs and inconvenience associated with more testing. You might also avoid being diagnosed with a health problem that would never harm you and avoid the psychological impacts like anxiety and depression that some people experience with more testing.

**Reference for threshold cut off:** American College of Cardiology 2017 Guideline for the prevention, detection, evaluation, and management of high blood pressure in adults Accessed February <https://www.acc.org/~/media/Non-Clinical/Files-PDFs-Excel-MS-Word-etc/Guidelines/2017/Guidelines_Made_Simple_2017_HBP.pdf>

**Pre-Hypertension Description Only**

Imagine you are 50, exercise a couple of times a week, try to eat well, and you feel healthy. You recently had a health check with your doctor. At this check up your blood tests came back with no problems, but your doctor observed that your blood pressure was a bit elevated. A current guideline rates your test level as close to the threshold for a potential problem.

Your doctor said you could have another test next week to test the stability of this reading, you could have another test later in the year, or you could choose not to have another test because your test level is close to the normal threshold. Alternatively, your doctor said to help lower your blood pressure and improve your health overall you could increase your exercise to ½ hour walks for 5 days a week, eat less salty foods and drink less alcohol.

Overdiagnosis occurs in medical practice when you get a diagnosis that ends up causing you more harm than good. It happens often when a healthy person is diagnosed with a very early form of a potential health problem, but that problem would never in fact have developed to cause them any symptoms or even any health problems. Having a slightly elevated blood pressure could increase your risk of developing a serious health problem in the next 5 years. However, 96% of people with similar blood pressure do not go on to have this problem within 5 years, so you have the potential to be overdiagnosed.

There is a risk that you may have a problem with your blood pressure. You could therefore go for further testing. Identifying a potential health problem early could slow progression towards a more serious problem if one was going to occur. If you choose not to take future tests, you would benefit by avoiding further costs and inconvenience associated with more testing. You might also avoid being diagnosed with a health problem that would never harm you and avoid the psychological impacts like anxiety and depression that some people experience with more testing.

**Reference for threshold cut off:** American College of Cardiology 2017 Guideline for the prevention, detection, evaluation, and management of high blood pressure in adults Accessed February <https://www.acc.org/~/media/Non-Clinical/Files-PDFs-Excel-MS-Word-etc/Guidelines/2017/Guidelines_Made_Simple_2017_HBP.pdf>

**Chronic Kidney Disease Labelled**

Imagine you are 65. You think you eat well, try to exercise daily and recently saw your doctor who checked your blood pressure and did some blood tests. Your blood pressure and cholesterol were fine, but your kidney function was 59 ml/min/1.73 m^2^ and your doctor said that you may have stage 3 Chronic Kidney Disease. A current guideline suggests that a test cut-off for this potential problem should be at or over 60 ml/min/1.73 m^2^. You are close to the threshold.

Your doctor said you could have another test next week to test the stability of this reading, you could have another test later in the year, or you could choose not to have another test because your test level is close to the normal threshold. Alternatively, your doctor said to help improve your kidney function and overall health, you could increase your exercise to ½ hour walks for 5 days a week, eat more dietary fibre, eat less refined sugars and flours and drink less alcohol.

Overdiagnosis occurs in medical practice when you get a diagnosis that ends up causing you more harm than good. It happens often when a healthy person is diagnosed with a very early form of a potential health problem, but that problem would never in fact have developed to cause them any symptoms or even any health problems. Having a problem with your kidney function could lead to a very slight chance (1%) you could develop a health problem that may result in needing dialysis. However, some studies suggest that at least 30% of people your age are classified as having this problem, but it never progresses to a serious concern. You have the potential to be overdiagnosed.

There is a risk that you may have a problem with your kidney function. You could therefore go for further testing. Identifying a potential health problem early could slow progression towards a more serious problem if one was going to occur. If you choose not to take future tests, you would benefit by avoiding further costs and inconvenience associated with more testing. You might also avoid being diagnosed with a health problem that would never harm you and avoid the psychological impacts like anxiety and depression that some people experience with more testing.

**Reference for threshold cut off:** Royal Australian College of General Practitioners (RACGP) Guidelines for preventive activities in general practice 9^th^ edition Accessed February 2018 <https://www.racgp.org.au/your-practice/guidelines/redbook/8-prevention-of-vascular-and-metabolic-disease/86-kidney-disease/>

**Chronic Kidney Disease Description Only**

Imagine you are 65. You think you eat well, try to exercise daily and recently saw your doctor who checked your blood pressure and did some blood tests. Your blood pressure and cholesterol were fine, but your kidney function was slightly low, and your doctor said it could be a potential problem with your kidneys. A current guideline suggests your test level is close to the threshold for a potential problem.

Your doctor said you could have another test next week to test the stability of this reading, you could have another test later in the year, or you could choose not to have another test because your test level is close to the normal threshold. Alternatively, your doctor said to help improve your kidney function and overall health, you could increase your exercise to ½ hour walks for 5 days a week, eat more dietary fibre, eat less refined sugars and flours and drink less alcohol.

Overdiagnosis occurs in medical practice when you get a diagnosis that ends up causing you more harm than good. It happens often when a healthy person is diagnosed with a very early form of a potential health problem, but that problem would never in fact have developed to cause them any symptoms or even any health problems. Having a problem with your kidney function could lead to a very slight chance (1%) you could develop a serious problem. However, some studies suggest that at least 30% of people your age are classified as having this problem, but it never progresses to a serious concern. You have the potential to be overdiagnosed.

There is a risk that you may have a problem with your kidney function. You could therefore go for further testing. Identifying a potential health problem early could slow progression towards a more serious problem if one was going to occur. If you choose not to take future tests, you would benefit by avoiding further costs and inconvenience associated with more testing. You might also avoid being diagnosed with a health problem that would never harm you and avoid the psychological impacts like anxiety and depression that some people experience with more testing.

**Reference for threshold cut off:** Royal Australian College of General Practitioners (RACGP) Guidelines for preventive activities in general practice 9^th^ edition Accessed February 2018 <https://www.racgp.org.au/your-practice/guidelines/redbook/8-prevention-of-vascular-and-metabolic-disease/86-kidney-disease/>

**Mild Hyperlipidaemia Labelled**

Imagine you are 50. You think you eat well, try to exercise daily and recently saw your doctor who checked your blood pressure and did some blood tests. Your blood pressure was fine but your doctor observed that your total cholesterol was 5.2mmol/L and your HDL cholesterol was 1mmol/L which your doctor said might suggest you have mild hyperlipidaemia (mildly high cholesterol). A current guideline suggests that a test cut-off for this potential problem should be less than 5.17mmol/L for total cholesterol and more than 1.0mmol/L for HDL cholesterol. You are close to the threshold.

Your doctor said you could have another test next week to test the stability of this reading, you could have another test later in the year, or you could choose not to have another test because your test level is close to the normal threshold. Alternatively, your doctor said to help improve your cholesterol and overall health, you could eat less red meat, and more low-fat dairy, olive oil and oily fish, and increase your exercise to ½ hour walks for 5 days a week.

Overdiagnosis occurs in medical practice when you get a diagnosis that ends up causing you more harm than good. It happens often when a healthy person is diagnosed with a very early form of a potential health problem, but that problem would never in fact have developed to cause them any symptoms or even any health problems. Having slightly elevated cholesterol could increase your risk of having a heart attack or stroke in the next 5 years. However, 97% of people like you do not go on to have these problems within 5 years. You have the potential to be overdiagnosed.

There is a risk that you may have a problem with cholesterol. You could therefore go for further testing. Identifying a potential health problem early could slow progression towards a more serious problem if one was going to occur. If you choose not to take future tests, you would benefit by avoiding further costs and inconvenience associated with more testing. You might also avoid being diagnosed with a health problem that would never harm you and avoid the psychological impacts like anxiety and depression that some people experience with more testing.

**Reference for threshold cut off:**  UpToDate website Accessed February 2018 <https://www.uptodate.com/contents/high-cholesterol-and-lipids-hyperlipidemia-beyond-the-basics>

**Mild Hyperlipidaemia Description Only**

Imagine you are 50. You think you eat well, try to exercise daily and recently saw your doctor who checked your blood pressure and did some blood tests. Your blood pressure was fine, but your doctor observed that your total cholesterol was slightly elevated which your doctor said might suggest you have a problem with how your body processes cholesterol. A current guideline rates your test results as close to the threshold for a potential problem.

Your doctor said you could have another test next week to test the stability of this reading, you could have another test later in the year, or you could choose not to have another test because your test level is close to the normal threshold. Alternatively, your doctor said to help improve your cholesterol and overall health, you could eat less red meat, and more low-fat dairy, olive oil and oily fish, and increase your exercise to ½ hour walks for 5 days a week.

Overdiagnosis occurs in medical practice when you get a diagnosis that ends up causing you more harm than good. It happens often when a healthy person is diagnosed with a very early form of a potential health problem, but that problem would never in fact have developed to cause them any symptoms or even any health problems. Having slightly elevated cholesterol could increase your risk of having a serious health problem in the next 5 years. However, 97% of people like you do not go on to have these problems within 5 years. You have the potential to be overdiagnosed.

There is a risk that you may have a problem with cholesterol. You could therefore go for further testing. Identifying a potential health problem early could slow progression towards a more serious problem if one was going to occur. If you choose not to take future tests, you would benefit by avoiding further costs and inconvenience associated with more testing. You might also avoid being diagnosed with a health problem that would never harm you and avoid the psychological impacts like anxiety and depression that some people experience with more testing.

**Reference for threshold cut off:**  UpToDate website Accessed February 2018 <https://www.uptodate.com/contents/high-cholesterol-and-lipids-hyperlipidemia-beyond-the-basics>

| S1 Table. Means and standard deviations for risk perception, stigma, intention to test, confidence and satisfaction across health conditions. | | | | | | | | | | | | | | | | | | | | | | | | | | | | | | | | | |  |
| --- | --- | --- | --- | --- | --- | --- | --- | --- | --- | --- | --- | --- | --- | --- | --- | --- | --- | --- | --- | --- | --- | --- | --- | --- | --- | --- | --- | --- | --- | --- | --- | --- | --- | --- |
|  |  | | Pre-diabetes Labelled  (n=64) | | | | Pre-diabetes Unlabelled (n=64) | | | | Pre-Hypertension Labelled  (n=69) | | | | Pre-Hypertension Unlabelled (n=63) | | | | Chronic Kidney Disease 3A Labelled  (n=61) | | | | Chronic Kidney Disease 3A Unlabelled (n=65) | | | | Mild Hyperlipideamia Labelled  (n=64) | | | | Mild Hyperlipideamia Unlabelled (n=62) | | | |
|  |  | | Mean | | SD | | Mean | | SD | | Mean | | SD | | Mean | | SD | | Mean | | SD | | Mean | | SD | | Mean | | SD | | Mean | | SD | |
| Risk Perception (score 1 - 7) | |  | |  | |  | |  | |  | |  | |  | |  | |  | |  | |  | |  | |  | |  | |  | |  | |  |
|  | Individual responsibility vs Bad luck | | 3.0 | | 1.5 | | 3.0 | | 1.5 | | 3.4 | | 1.3 | | 3.1 | | 1.2 | | 3.3 | | 1.2 | | 3.6 | | 1.3 | | 3.0 | | 1.3 | | 2.9 | | 1.2 | |
|  | Known vs Unknown risks | | 3.4 | | 1.6 | | 3.6 | | 1.8 | | 3.8 | | 1.7 | | 3.7 | | 1.5 | | 3.6 | | 1.6 | | 4.2 | | 1.6 | | 3.6 | | 1.5 | | 3.7 | | 1.5 | |
|  | Common vs Dreaded Condition | | 3.3 | | 1.5 | | 3.2 | | 1.4 | | 3.8 | | 1.5 | | 3.1 | | 1.3 | | 3.2 | | 1.3 | | 3.4 | | 1.1 | | 3.1 | | 1.5 | | 2.8 | | 1.3 | |
|  | Immediate vs Delayed risk | | 4.6 | | 1.5 | | 4.5 | | 1.7 | | 4.4 | | 1.6 | | 4.6 | | 1.3 | | 4.8 | | 1.5 | | 4.6 | | 1.6 | | 4.8 | | 1.5 | | 4.9 | | 1.6 | |
|  | Uncontrollable vs Controllable Condition | | 5.7 | | 1.3 | | 5.8 | | 1.2 | | 5.2 | | 1.3 | | 5.2 | | 1.3 | | 5.7 | | 1.2 | | 4.9 | | 1.3 | | 5.7 | | 1.2 | | 5.6 | | 1.4 | |
|  | Unconcerned vs Concerned | | 4.4 | | 1.6 | | 4.6 | | 1.3 | | 4.6 | | 1.5 | | 4.0 | | 1.4 | | 4.4 | | 1.1 | | 4.4 | | 1.4 | | 4.2 | | 1.7 | | 4.2 | | 1.4 | |
| Stigma  (score 1 - 7) | |  | |  | |  | |  | |  | |  | |  | |  | |  | |  | |  | |  | |  | |  | |  | |  | |  |
|  | Comfort | | 5.6 | | 1.4 | | 5.3 | | 1.4 | | 5.4 | | 1.4 | | 5.0 | | 1.6 | | 5.7 | | 1.4 | | 5.1 | | 1.5 | | 5.4 | | 1.5 | | 5.7 | | 1.3 | |
|  | Embarrassment | | 2.3 | | 1.4 | | 2.5 | | 1.7 | | 2.2 | | 1.6 | | 2.2 | | 1.5 | | 2.1 | | 1.3 | | 2.3 | | 1.3 | | 1.9 | | 1.3 | | 2.1 | | 1.4 | |
|  | Social Isolation | | 2.4 | | 1.7 | | 2.4 | | 1.5 | | 2.8 | | 1.7 | | 2.4 | | 1.5 | | 2.2 | | 1.5 | | 2.7 | | 1.6 | | 2.1 | | 1.4 | | 2.2 | | 1.5 | |
|  | Concealment | | 5.4 | | 1.7 | | 5.2 | | 1.7 | | 5.4 | | 1.3 | | 5.1 | | 1.6 | | 5.7 | | 1.5 | | 5.0 | | 1.6 | | 5.8 | | 1.4 | | 5.7 | | 1.5 | |
| Intention to conduct future test (score 1 - 10) | | 7.0 | | 2.7 | | 7.4 | | 2.3 | | 7.4 | | 2.6 | | 7.3 | | 2.3 | | 7.1 | | 2.8 | | 7.5 | | 2.1 | | 6.7 | | 2.6 | | 6.9 | | 2.4 | |  |
| Confidence in decision  (score 1 - 7) | | 8.0 | | 1.9 | | 8.0 | | 2.0 | | 8.1 | | 1.8 | | 7.7 | | 2.0 | | 8.5 | | 1.7 | | 7.8 | | 2.1 | | 7.6 | | 2.2 | | 7.6 | | 2.2 | |  |
| Satisfaction in decision  (score 1 - 7) | | 8.0 | | 1.9 | | 7.9 | | 1.9 | | 8.2 | | 1.8 | | 7.8 | | 1.9 | | 8.3 | | 1.9 | | 8.0 | | 2.0 | | 7.8 | | 2.0 | | 7.7 | | 2.1 | |  |

| **S2 Table. Rotated component matrix for perceptual map** | | | | |
| --- | --- | --- | --- | --- |
|  |  | Component | | |
|  |  | Factor 1 | Factor 2 | Factor 3 |
| Risk Perception Measures | |  |  |  |
|  | Responsibility vs Luck | -0.15 | 0.73 | -0.33 |
|  | Known vs Unknown risks | 0.13 | 0.78 | 0.20 |
|  | Common vs Dreaded Condition | 0.73 | 0.35 | -0.22 |
|  | Immediate vs Delayed risk | -0.52 | 0.49 | 0.50 |
|  | Uncontrollable vs Controllable Condition | -0.07 | -0.05 | 0.91 |
|  | Unconcerned vs Concerned | 0.84 | -0.19 | 0.06 |
| Extraction Method: Principal Component Analysis. | | | | |
| Rotation Method: Varimax with Kaiser Normalization. | | | | |

| **S3 Table. Mean factor scores by health condition scenario** | | | | |
| --- | --- | --- | --- | --- |
|  |  | Factor 1 | Factor 2 | Factor 3 |
|  | Pre-diabetes (L) | 0.08 | -0.01 | 0.15 |
|  | Pre-diabetes (NL) | 0.05 | 0.06 | 0.18 |
|  | Pre-hypertension (L) | 0.13 | 0.06 | 0.04 |
|  | Pre-hypertension (NL) | -0.11 | -0.09 | -0.25 |
|  | Chronic kidney disease 3a (L) | 0.11 | 0.06 | -0.02 |
|  | Chronic kidney disease 3a (NL) | 0.00 | 0.20 | -0.20 |
|  | Mild Hyperlipidaemia (L) | -0.04 | -0.19 | -0.01 |
|  | Mild hyperlipidaemia (NL) | -0.23 | -0.10 | 0.12 |
| Note: L = label, NL = description only (no label) | | | |  |

**Survey conducted in Qualtrics**

15 March 2018

RO 016123

My name is Rae Thomas. I am an Assistant Professor in the Faculty of Health Sciences and Medicine at Bond University. My colleagues and I are conducting research that investigates how people, like you, perceive various health scenarios. There are no right or wrong answers – we are just interested in your opinions.

As part of this study, we invite you to complete an on-line questionnaire that will take approximately 20 to 30 minutes. Your participation is completely voluntary. You will be asked to respond to questions about yourself and health scenarios. If this causes you distress, you may discontinue the survey at any time without any negative consequences. We will not collect any personal identifying information, so your responses will be completely anonymous. If you choose to withdraw from participating in this study, the information you have provided will be immediately destroyed.

Data will be stored in a secured location at Bond University for a period of five years in accordance with Bond University Human Research Ethics Committee guidelines. Should you have any complaints concerning the manner in which this research is being conducted please make contact:

Bond University Human Research Ethics Committee Bond University Office of Research Services.

Bond University, Gold Coast, 4229, Australia

Tel: +61 7 5595 4194

Fax: +61 7 5595 1120

email: [ethics@bond.edu.au](mailto:ethics@bond.edu.au)

Thank you for taking the time to assist us with this important research.

Yours sincerely,

Rae Thomas

By clicking "Continue to Survey" you are indicating your consent for participation.

o Continue to Survey

We care about the quality of our survey data and hope to receive the most accurate measures of your opinions, so it is important to us that you thoughtfully provide your best answer to each question in the survey.

Do you commit to providing your thoughtful and honest answers to the questions in this survey?

o I will provide my best answers

o I will not provide my best answers

o I can't promise either way

In which country do you currently reside?

(Full range of options provided, and participants excluded if not residing in either Australia, Canada and Ireland)

What gender do you most identify with?

o Male

o Female

o Other ________________________________________________

What is your age?

________________________________________________________________

This part of the survey is about understanding your overall attitude towards health. Please rate each item as it relates to you.

|  | Strongly Disagree (1) | Disagree  (2) | Somewhat Disagree (3) | Somewhat Agree  (4) | Agree  (5) | Strongly Agree (6) |
| --- | --- | --- | --- | --- | --- | --- |
| 1. If my health worsens, it is my own behaviour which determines how soon I will feel better again. |  |  |  |  |  |  |
| 2. As to my health, what will be will be. |  |  |  |  |  |  |
| 3. If I see my doctor regularly, I am less likely to have problems with my health. |  |  |  |  |  |  |
| 4. Most things that affect my health happen to me by chance. |  |  |  |  |  |  |
| 5. Whenever my health worsens, I should consult a medically trained professional. |  |  |  |  |  |  |
| 6. I am directly responsible for my health getting better or worse. |  |  |  |  |  |  |
| 7. Other people play a big role in whether my health improves, stays the same, or gets worse. |  |  |  |  |  |  |
| 8. Whatever goes wrong with my health is my own fault. |  |  |  |  |  |  |
| 9. Luck plays a big part in determining how my health improves. |  |  |  |  |  |  |
| 10. In order for my health to improve, it is up to other people to see that the right things happen. |  |  |  |  |  |  |
| 11. Whatever improvement occurs with my health is largely a matter of good fortune. |  |  |  |  |  |  |
| 12. The main thing which affects my health is what I myself do. |  |  |  |  |  |  |
| 13. I deserve the credit when my health improves and the blame when it gets worse. |  |  |  |  |  |  |
| 14. Following doctor's orders to the letter is the best way to keep my health from getting any worse. |  |  |  |  |  |  |
| 15. If my health worsens, it's a matter of fate. |  |  |  |  |  |  |
| 16. If I am lucky, my health will get better. |  |  |  |  |  |  |
| 17. If my health takes a turn for the worse, it is because I have not been taking proper care of myself. |  |  |  |  |  |  |
| 18. The type of help I receive from other people determines how soon my health improves. |  |  |  |  |  |  |

The following questions are about behaviour in general. Please rate each item as it relates to you.

|  | Strongly Disagree (1) | Disagree  (2) | Somewhat Disagree (3) | Somewhat Agree  (4) | Agree  (5) | Strongly Agree (6) |
| --- | --- | --- | --- | --- | --- | --- |
| 1. When it comes to achieving things that are important to me, I find that I don't perform as well as I would ideally like to. |  |  |  |  |  |  |
| 2. I feel I have made progress toward being successful in my life. |  |  |  |  |  |  |
| 3. When I see an opportunity for something I like, I get excited right away. |  |  |  |  |  |  |
| 4. I frequently imagine how I will achieve my hopes and aspirations. |  |  |  |  |  |  |
| 5. I see myself as someone who is primarily striving to reach my "ideal self"- to fulfil my hopes, wishes and aspirations. |  |  |  |  |  |  |
| 6. I usually obeyed rules and regulations that were established by my parents. |  |  |  |  |  |  |
| 7. Not being careful enough has gotten me into trouble at times. |  |  |  |  |  |  |
| 8. I worry about making mistakes. |  |  |  |  |  |  |
| 9. I frequently think about how I can prevent failures in my life. |  |  |  |  |  |  |
| 10. I see myself as someone who is primarily striving to become the self I "ought" to be –to fulfil my duties, responsibilities and obligations. |  |  |  |  |  |  |

How would you rate your overall health?

| Very Poor |  |  |  |  |  |  |  |  | Very Good |
| --- | --- | --- | --- | --- | --- | --- | --- | --- | --- |
| 1 | 2 | 3 | 4 | 5 | 6 | 7 | 8 | 9 | 10 |

The following questions are about how you approach health and medical care. Please rate

each item as it relates to you.

|  | Strongly Disagree  (1) | Disagree  (2) | Somewhat Disagree  (3) | Neither disagree nor agree (4) | Somewhat Agree  (5) | Agree  (6) | Strongly Agree  (7) |
| --- | --- | --- | --- | --- | --- | --- | --- |
| 1. It is important to treat disease even when it does not make a difference in survival. |  |  |  |  |  |  |  |
| 2. It is important to treat a disease even when it does not make a difference in quality of life. |  |  |  |  |  |  |  |
| 3. Doing everything to fight illness is always the right choice. |  |  |  |  |  |  |  |
| 4. When it comes to health care, the only responsible thing to do is to actively seek medical care. |  |  |  |  |  |  |  |
| 5. If I have a health issue, my preference is to wait and see if the problem gets better on its own before going to the doctor. |  |  |  |  |  |  |  |
| 6. If I feel unhealthy, the first thing that I do is to go to the doctor and get a prescription. |  |  |  |  |  |  |  |
| 7. I often suggest that friends and family see their doctor. |  |  |  |  |  |  |  |
| 8. When it comes to health care, watching and waiting is never an acceptable option. |  |  |  |  |  |  |  |
| 9. If I have a medical problem, my preference is to go straight to a doctor and ask his or her opinion. |  |  |  |  |  |  |  |
| 10. When it comes to medical treatment, more is usually better. |  |  |  |  |  |  |  |

The next few questions try to understand you as a person. I see myself as:

|  | Strongly Disagree  (1) | Disagree  (2) | Somewhat Disagree  (3) | Neither disagree nor agree  (4) | Somewhat Agree  (5) | Agree  (6) | Strongly Agree  (7) |
| --- | --- | --- | --- | --- | --- | --- | --- |
| 1. Extraverted, enthusiastic |  |  |  |  |  |  |  |
| 2. Critical, quarrelsome |  |  |  |  |  |  |  |
| 3. Dependable, self-disciplined |  |  |  |  |  |  |  |
| 4. Anxious, easily upset |  |  |  |  |  |  |  |
| 5. Open to new experiences, complex |  |  |  |  |  |  |  |
| 6. Reserved, quiet |  |  |  |  |  |  |  |
| 7. Sympathetic, warm |  |  |  |  |  |  |  |
| 8. Disorganised, careless |  |  |  |  |  |  |  |
| 9. Calm, emotionally stable |  |  |  |  |  |  |  |
| 10. Conventional, uncreative |  |  |  |  |  |  |  |

The next battery of questions is about your behaviour. Please rate the likelihood of you doing the following:

|  | Extremely Unlikely  (1) | Moderately Unlikely  (2) | Not Sure  (3) | Moderately Likely  (4) | Extremely Likely  (5) |
| --- | --- | --- | --- | --- | --- |
| 1. Consuming five or more servings of alcohol in a single evening. |  |  |  |  |  |
| 2. Walking home alone at night in a somewhat unsafe area of town. |  |  |  |  |  |
| 3. Regularly eating high cholesterol foods. |  |  |  |  |  |
| 4. Ignoring some persistent physical pain by not going to the doctor. |  |  |  |  |  |
| 5. Taking a medical drug that has a high likelihood of negative side effects. |  |  |  |  |  |

**Two scenarios of either labelled or description only provided here and the following questions were asked after each scenario.**

To what extent do you feel the individual is responsible for having this problem, or do you think the health problem was entirely due to bad luck?

| Individual Responsible |  |  |  |  |  | Due to Bad Luck |
| --- | --- | --- | --- | --- | --- | --- |
| 1 | 2 | 3 | 4 | 5 | 6 | 7 |

To what extent are the risks precisely known by a person that has this problem?

| Known Precisely |  |  |  |  |  | Not Known Precisely |
| --- | --- | --- | --- | --- | --- | --- |
| 1 | 2 | 3 | 4 | 5 | 6 | 7 |

Does this problem present a risk that people have learned to live with and can think about reasonably calmly, or is it one that people have great dread for—on the level of a gut reaction?

| Common Condition |  |  |  |  |  | Dreadful Condition |
| --- | --- | --- | --- | --- | --- | --- |
| 1 | 2 | 3 | 4 | 5 | 6 | 7 |

For the problem presented, to what extent is the risk to the persons health immediate—or is any risk likely to occur at some later time?

| Risks are Immediate |  |  |  |  |  | Risks are Delayed |
| --- | --- | --- | --- | --- | --- | --- |
| 1 | 2 | 3 | 4 | 5 | 6 | 7 |

If you are exposed to the problem presented, to what extent can you, by personal skill or diligence avoid death or injury?

| It is out of my Control |  |  |  |  |  | It is Controllable |
| --- | --- | --- | --- | --- | --- | --- |
| 1 | 2 | 3 | 4 | 5 | 6 | 7 |

Please indicate how far you are concerned about the risks associated with this problem.

| Not at all Concerned |  |  |  |  |  | Extremely Concerned |
| --- | --- | --- | --- | --- | --- | --- |
| 1 | 2 | 3 | 4 | 5 | 6 | 7 |

To what extent would you feel comfortable being seen in public with this problem?

| Extremely Uncomfortable |  |  |  |  |  | Extremely Comfortable |
| --- | --- | --- | --- | --- | --- | --- |
| 1 | 2 | 3 | 4 | 5 | 6 | 7 |

To what extent would you associate this problem with shame or embarrassment?

| Not at all Likely |  |  |  |  |  | Very Likely |
| --- | --- | --- | --- | --- | --- | --- |
| 1 | 2 | 3 | 4 | 5 | 6 | 7 |

For this problem, to what extent do you think you would experience problems in forming social relationships?

| Not at all Likely |  |  |  |  |  | Very Likely |
| --- | --- | --- | --- | --- | --- | --- |
| 1 | 2 | 3 | 4 | 5 | 6 | 7 |

To what extent can this problem be concealed from others?

| Not at all Concealable |  |  |  |  |  | Very Concealable |
| --- | --- | --- | --- | --- | --- | --- |
| 1 | 2 | 3 | 4 | 5 | 6 | 7 |

Which best describes your intention to have the follow-up tests described in the scenario next week?

| Definitely will not |  |  |  |  |  |  |  |  | Definitely Will |
| --- | --- | --- | --- | --- | --- | --- | --- | --- | --- |
| 1 | 2 | 3 | 4 | 5 | 6 | 7 | 8 | 9 | 10 |

How confident are you with your decision to have the follow-on test?

| Not at all Confident |  |  |  |  |  |  |  |  | Very Confident |
| --- | --- | --- | --- | --- | --- | --- | --- | --- | --- |
| 1 | 2 | 3 | 4 | 5 | 6 | 7 | 8 | 9 | 10 |

How satisfied are you with your decision to have the follow-on test?

| Not at all Satisfied |  |  |  |  |  |  |  |  | Very Satisfied |
| --- | --- | --- | --- | --- | --- | --- | --- | --- | --- |
| 1 | 2 | 3 | 4 | 5 | 6 | 7 | 8 | 9 | 10 |

**Feedback**

We thank you for your time spent taking this survey.

Your response has been recorded.

Do you have any feedback or recommendations for our survey?

____________________________________________________________________________________________________________________________________________________________________________________________________________________________________________________________________________________________________________
